# The important challenge of quantifying tropical diversity

**DOI:** 10.1186/s12915-017-0358-6

**Published:** 2017-03-07

**Authors:** Anne E. Magurran

**Affiliations:** Centre for Biological Diversity, School of Biology, University of St Andrews, St Andrews, Scotland UK

## Abstract

The tropics are the repository of much of the world’s biodiversity, yet are undersampled relative to temperate regions. To help fill this knowledge gap, a paper in *BMC Biology* explores diversity patterns in tropical African plants, as revealed by the RAINBIO database. The paper documents spatial variation in diversity and data coverage, but also highlights the challenges faced in quantifying diversity patterns using data collated from a range of sources including herbaria.

See research article: http://bmcbiol.biomedcentral.com/articles/10.1186/s12915-017-0356-8.

## Commentary

**Fig. 1.**
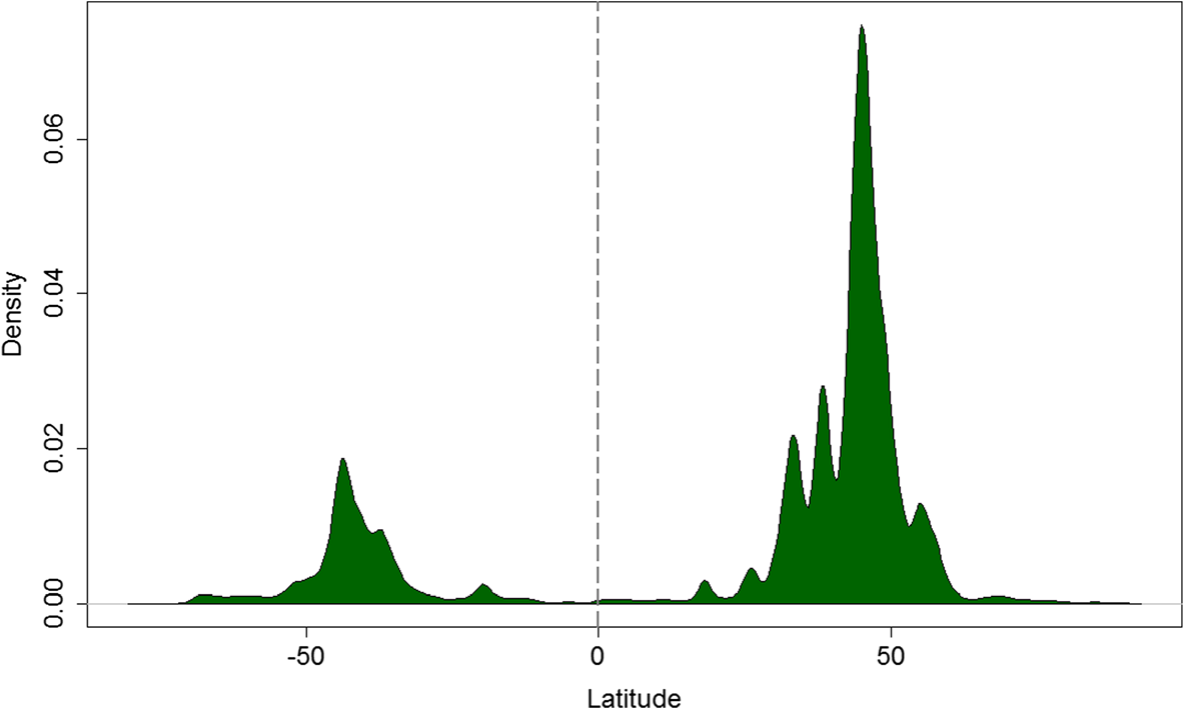
Rigorous ecological survey data are strongly concentrated in temperate latitudes. For example, this plot illustrates the density of survey points, in relation to latitude (degrees north and south, with the equator indicated by the *dashed line*), in the BioTIME database of assemblage time series [1]. The dearth of these data from tropical regions demonstrates the importance of databases [5] and analyses, as in [4], which draw on different data sources

The far-reaching impacts that our human species is having on the Earth’s ecosystems have led scientists to call the present era the Anthropocene. There can be no doubt that the world’s biodiversity is under unprecedented threat. Species extinctions make headline news, while natural communities are being reorganized at a rate that far exceeds historical baselines [1]. Yet, despite growing concern about the fate of the biosphere, substantial knowledge gaps with respect to the distribution and status of species remain. Most ecologists and taxonomists are based in temperate regions [2], which are also the most comprehensively surveyed. Large swathes of the Earth, particularly biodiverse tropical regions, are very poorly documented [3] (Fig. 1). A new paper, by Sosef et al. [4], reporting a synthesis of diversity patterns in tropical African plants [5], is thus an important and timely contribution.

The gold standard for biodiversity assessment is representative sampling using consistent and appropriate methodologies [6]. Robust field sampling demands expert taxonomic knowledge and the ability to sample localities selected using stratified random sampling [6]. Unfortunately, in many parts of the world, the resources needed for such surveys are limited or absent, while the terrain within which these poorly documented assemblages occur can be unsafe or inaccessible. Citizen science is often hailed as a means of plugging knowledge gaps but it too is concentrated in regions that are already relatively well documented. Researchers therefore need to turn to other sources of evidence, including information gleaned from specimens deposited in museums and herbaria, *ad hoc* inventories, and personal records, as exemplified in the RAINBIO resource [5], a curated database of vascular plant species in tropical Africa.

The analysis of RAINBIO data by Sosef and colleagues [4] seeks to understand how plant diversity is distributed across tropical Africa. In this it demonstrates the challenges, and also the opportunities, afforded by databases assembled from different sources. One of Sosef et al.’s goals is to estimate species richness at two levels of spatial resolution: 0.5° grid cells and country level. To do this the investigators employ a non-parametric species richness estimator developed by Anne Chao (see [7]). In essence this measure, known as Chao 1, weights the number of observed species by the fraction recorded as either singletons (one specimen) or doubletons (two specimens). As the relative frequency of singletons increases, so too does the estimate of richness, the logic being that the observation of many rare taxa is indicative of a large number of unseen species. Chao’s estimator provides a minimum estimate of richness and assumes homogeneity amongst samples [8]. This latter expectation is likely to be violated in databases constructed from a range of sources. Indeed, investigations in other tropical ecosystems (for example, [3]) highlight the extent to which variation in sampling effort biases species richness estimation. Sosef et al. [4] find, as did Engemann and colleagues [3], that estimated richness correlates with sampling effort. Since herbaria and museums value breadth and rarity in their collections, databases populated from these sources are prone to over-emphasizing singleton and doubleton species. However, it is worth noting that rigorous surveys of tropical ecosystems similarly report high numbers of singleton taxa [9].

Other metrics employed in the paper, such as the Neilsen diversity metric, are less sensitive to sampling effort than richness estimators. Nonetheless, it is important to remember that the value the Neilsen statistic calculates—dubbed ‘effective species’—refers to an attribute of the data set and does not necessarily provide an ecologically meaningful insight into the structure of the assemblages from which the data were sourced. Even turnover metrics, which evaluate compositional change over space or time and might seem to be less obviously affected by sampling effort, can be influenced by inconsistencies in sampling [8].

These caveats could suggest that analyses based on heterogeneous databases are so fraught with problems as to be uninformative. Yet, provided that the results are interpreted cautiously, such analyses can provide a crucial initial step towards a better understanding of diversity patterns and help steer future investigations towards productive quests. The first contribution of these analyses is that they make plain the extent of heterogeneity in data collection over space and time. Although collecting dates in RAINBIO range from 1782 to 2015, collecting intensity is not constant. For instance, recording in the Democratic Republic of Congo occurred mostly from the 1930s to the 1960s [4]. Clear recommendations for improving understanding of tropical diversity, such as improved data exchange between datasets, are identified.

A second contribution of analyses of databases such as RAINBIO is that firmer estimates of richness, endemicity, range extent and patterns of commonness and rarity begin to emerge. While is it wise to be careful about estimates of richness and reported values of diversity statistics, especially when making comparisons with other regions and systems, these results help shape hypotheses and guide objectives for future studies. For example, Sosef and colleagues’ [4] analysis of diversity patterns and data coverage is well placed to inform new investigations on a range of topics, such as the consequences for biodiversity of climate change, and to highlight the groups and regions in which taxonomic research is most urgently needed.

New insights into functional diversity represent a third contribution of an analysis such as this [4]. Sosef et al. report geographic variation in plant growth form dominance across tropical Africa, a field of research in which data have previously been scarce. This advance is possible because the observed diversity patterns are underpinned by georeferenced data on named species.

Finally, a macroecological analysis such as Sosef et al.’s, can be viewed alongside other investigations of data with a similar geographical scope, for example Marshall et al.’s [10] recent assessment of rarity amongst the tropical African flora. Of course different compilations of data may share many of the same shortcomings, not least because some overlap in source material is likely, but as long as they are not interpreted too simplistically, complementary analyses such as Sosef et al.’s [4] and Marshall et al.’s [10] jointly advance understanding and are well placed to support the conservation of biodiversity in our rapidly changing world. In particular they underline the pressing need for better understanding of the distribution and nature of tropical diversity. Innovative and insightful analyses of data in RAINBIO and other databases will be key to achieving this aim.
